# Chronic Rhinosinusitis—Microbiological Etiology, Potential Genetic Markers, and Diagnosis

**DOI:** 10.3390/ijms25063201

**Published:** 2024-03-11

**Authors:** Michał Michalik, Beata Krawczyk

**Affiliations:** 1 Medical Center MML, Bagno 2, 00-112 Warsaw, Poland; m.michalik@mml.com.pl; 2 Department of Biotechnology and Microbiology, Faculty of Chemistry, Gdańsk University of Technology, G. Narutowicza 11/12, 80-233 Gdańsk, Poland

**Keywords:** sinusitis, predisposition to CRS, microbiota, diagnostic methods, problem of susceptibility to antibiotics, probiotics

## Abstract

Chronic rhinosinusitis (CRS) is a significant public health problem. Bacterial colonization and impaired mucociliary clearance play a significant role in the inflammatory process. Several inflammatory pathways and host defense elements are altered in CRS, which may contribute to observed differences in the microbiome. To date, researching CRS has been difficult due to limited access to the studied tissue and a lack of available biomarkers. Ongoing scientific research is increasingly based on simple and objective analytical methods, including sensors, detection with PCR, and sequencing. Future research on microbiota and human factors should also include genomics, transcriptomics, and metabolomics approaches. This report analyzes the changes that occur in the paranasal sinuses of people with acute and chronic rhinosinusitis, the composition of the microbiota, the human genetic markers that may shed light on the predisposition to CRS, and the advantages and disadvantages of classical and molecular diagnostic methods, as well as addressing the difficulties of sinusitis treatment.

## 1. Introduction—Sinus Function, Immunology of Healthy Sinuses

The paranasal sinuses are pneumatic spaces in the facial bones that connect to the nasal cavity via narrow openings. There are frontal, maxillary, sphenoid, and ethmoid sinuses. The maxillary sinuses are the largest sinuses, located below the eye sockets, above the maxilla, symmetrically on both sides of the nose. The frontal sinuses are located between the two laminae of the frontal bone. They fully develop in the second decade of life. Sphenoid sinuses are small structures that resemble the shape of a butterfly’s wings. These sinuses are located deepest inside the facial cranium. The location of the sphenoid sinuses is the sphenoid bone, at eye level. The ethmoid sinuses are the smallest of all the sinuses. They are formed by six to ten ethmoid cells, meaning small cavities, which are lined with mucous membranes on the inside and divided by thin bony lamellae [1].

The paranasal sinuses perform numerous functions. First of all, they purify, heat, and humidify the inhaled air, and they equalize the pressure difference during breathing. In addition, they protect the skull from injury, as they create voids in the bones of the skull. During trauma, the damaged skull bones first enter the sinus space. Furthermore, the sinuses provide a layer of thermal insulation, warm the base of the skull and orbit, and result in a weight reduction in the craniofacial skeleton. The sinuses also act as a buffer, a resonating space that protects the inner ear [2].

Functioning sinuses are part of the immune response. They protect the body from the entry of viruses, bacteria, and other microorganisms. The nasal cavity and paranasal sinus tissues are exposed to airborne environmental agents, including pathogenic and non-pathogenic bacteria, viruses, fungi, allergens, and toxins. The surface mucosa uses several immune mechanisms to maintain homeostasis. Numerous factors affect the functionality of the immune response, which is believed to predispose individuals to develop chronic rhinosinusitis (CRS) [3].

The first line of defense against microorganisms and airborne particles is innate immunity. Innate immunity classically refers to non-specific defense mechanisms rapidly activated after exposure to antigenic material and providing immediate protection. It involves a physical barrier in the upper respiratory tract, provided by the ciliary respiratory epithelium lining the sinonasal cavity. This barrier contains scattered cup cells that secrete a layer of mucus that covers the epithelial surface. The mucus layer promotes the non-specific removal of microorganisms and irritant particles by the mucociliary mechanism. Barrier dysfunction, coupled with disruption of the mucociliary clearance mechanism, can contribute to the development of CRS. Genetic defects in ciliary function, as found in cystic fibrosis and primary ciliary dyskinesia, as well as acquired ciliary dysfunction, may also contribute to the aforementioned disorders [3].

Nasal sinus epithelial cells secrete enzymes, opsonins, defensins, permeabilizing proteins, and other endogenous antimicrobial products into the apical mucus layer. These host defense molecules are important for the direct neutralization of microorganisms and the recruitment of inflammatory cells that modulate the immune response. In addition, proteins such as lactoferrin, mucins, C-reactive protein, and secretory leukocyte proteinase inhibitor (SLPI) collectively provide protection against bacteria, fungi, and viruses. When pathogenic microorganisms invade the nasal sinus epithelium, circulating phagocytes recognize pathogen-associated molecular patterns (PAMPs) and damage-associated molecular patterns (DAMPs). Inflammatory pathways are activated as a result of the recognition and binding of host epithelial cells to pathogenic or damaged cellular proteins [4]. The literature data suggest that chronic inflammation may be caused by an abnormal immune response of the host mucosa rather than solely the presence of pathogens. In mucosal homeostasis, local irritants and pathogens are quickly and effectively eliminated through innate mucosal immune pathways without broader stimulation of the acquired immune system. The persistent inflammation seen in CRS may be due to a pathological imbalance in innate immune interactions between the host and the environment. Impaired critical innate immune protection makes the surface of the nasal mucosa vulnerable to colonization and potential damage, stimulating an adaptive (specific) immune response [3].

The endotypic classification of CRS mainly reflects the inflammatory mechanisms present in an individual [5]. Endotypes are often defined by the presence or absence of one or more biomarkers. The use of biomarkers can be helpful in making an accurate diagnosis, evaluating the optimal therapeutic strategy, and determining a patient’s prognosis. It is believed that CRS without the presence of nasal polyps is characterized by an inflammatory pattern with a predominance of T helper (Th) 1 cells, and CRS with the presence of nasal polyps is characterized by an inflammatory pattern with a predominance of Th2 cells. Th cells are a subpopulation of T lymphocytes with functions related to immune response stimulation. Th1 lymphocytes are responsible for the body’s cellular (cell-mediated immunity) response, while Th2 cells are responsible for the humoral (antibody-mediated immunity) response. However, the literature data have shown that CRS is an inflammatory process with variable and non-mutually exclusive immune markers [6].

## 2. Changes That Occur in the Sinuses of Humans with Acute and Chronic Rhinosinusitis

The sinus mucosa is a continuous structure with the nasal cavity, such that any infection of the nasal mucosa can easily spread to the sinuses. The mucous membrane of the sinuses produces large amounts of mucus secretions that enable the removal of contaminants using a mucociliary mechanism. The swinging movement of the cilia eliminates secretions from the sinuses to the outside [7]. CRS is an inflammatory process. Swelling of the nasal mucosa is a consequence of inflammation. As a result of mucosal edema, mucociliary movement is dysfunctional, and secretion outflow is impaired [8]. The collected secretions become a medium for bacterial growth. There are perfect conditions (temperature, humidity) for the development of bacterial microbiota in the sinuses; therefore, the resident bacterial strains, both saprophytic and potentially pathogenic, multiply over a short time [9]. It is widely believed that allergic diseases, especially IgE-dependent inflammatory processes such as allergic rhinitis, are a factor that contributes to the development of CRS or a comorbid disease/factor accompanying the spread of CRS [10]. Song et al. reported that the rate of sinusitis was 3.1 times higher in people with allergy symptoms than in people without allergy symptoms (22.4 vs. 7.1%) [11].

Rhinosinusitis is defined clinically as inflammation characterized by the presence of two or more symptoms, one of which should be nasal blockage/obstruction, nasal congestion or discharge, and/or facial pain/squeezing and decreased smell or loss of smell, in combination with objective signs of disease identified by endoscopy or CT scan. Acute sinusitis is diagnosed if symptoms have an acute onset and last less than twelve weeks. Symptoms lasting longer than twelve weeks are the basis for the diagnosis of CRS [3].

Studies have confirmed that CRS is a multifactorial condition. The predisposition of the host to develop the condition plays a key role, and a variety of external factors can potentially trigger or modify the disease in a predisposed individual. An increasingly common hypothesis is that innate immune defects, which disrupt normal mucosal homeostasis and allow microorganisms and airborne particles to stimulate the immune response, are responsible for the incidence of CRS. The further failure of innate immune mechanisms to regulate inflammation and restore homeostasis can result in chronic inflammation, even in the absence of a trigger [3].

For the purpose of discussing the clinical trials, CRS was divided into two broad categories based on the presence or absence of nasal polyps. In CRS with nasal polyps (CRSwNP), inflammation is typically eosinophilic in nature and is associated with a type 2 T helper (Th) cytokine profile that is not evident in CRS without nasal polyps (CRSsNP). The second type of CRS is usually dominated by Th1 cytokines. These are not clear-cut divisions; both forms of CRS present a mixed Th1/Th2 profile [3].

According to new guidelines from the European Position Paper on Rhinosinusitis and Nasal Polyps 2020 (EPOS2020), the basic definition of CRS and the time criteria did not change: symptoms ≤ 12 weeks and an incomplete resolution of symptoms [12]. However, the division of CRS changed significantly, and this classification takes into account local anatomical factors associated with pathogenesis and endotype dominance for primary CRS. The CRS classification is based on the concepts of phenotype, i.e., the clinical picture, triggering factors, and inflammatory parameters and endotype, which is the result of genetic and environmental factors (so-called individual characteristics), e.g., increased IgE, IL-5, and eosinophilia affecting the phenotype [13].

**Figure 1:**
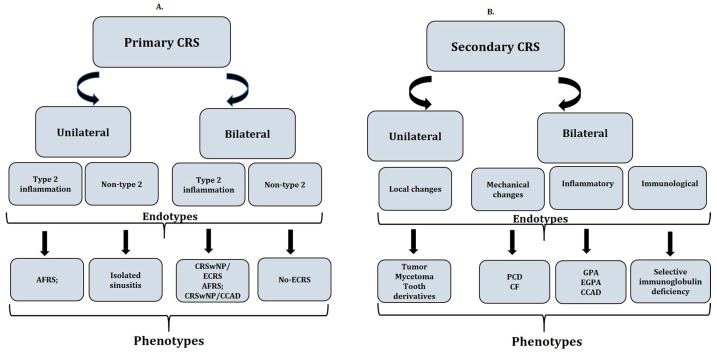
Classification of CRS based on the latest consensus (EPOS2020) [12]. (**A**). Classifying primary CRS taking into account endotypes and phenotypes. (**B**). Classifying secondary CRS taking into account endotypes and phenotypes. Legend: AFRS—allergic fungal rhinosinusitis; CRSwNP—CRS with nasal polyps; ECRS—eosinophilic chronic rhinosinusitis; CF—cystic fibrosis; PCD—primary ciliary dyskinesia; CCAD—central compartment atopic disease; GPA—granulomatosis with polyangiitis, (known as Wegener’s granulomatosis, WG); EGPA—eosinophilic granulomatosis with polyangiitis, formerly Churg-Strauss Syndrome.

Besides these divisions, according to EPOS2020, chronic sinusitis was subdivided into primary and secondary and into unilateral and bilateral, according to the anatomical location of the lesions. According to the predominant endotype, primary CRS was subdivided into those in which type 2 inflammation predominates and those in which there are no markers of type 2 inflammation (non-type 2) (Figure 1A). Type 2 nasal obstruction comprises a loss of smell, asthma, atopy (allergy), NSAID intolerance [NSAID-enhanced respiratory disease (N-ERD)]; in endoscopy—polyps and “eosinophilic” secretion (stringy, sticky, yellowish); and in tests—eosinophilia and increased IgE. Non-type 2 is characterized by discharge (also post nasal drip), pain, and less often asthma and atopy; in endoscopy—a predominance of often purulent-colored secretion and swelling; and in tests—no eosinophilia and IgE within the norm.

Several factors have been hypothesized to play a role in CRS, including chronic mucosal inflammation secondary to mucociliary clearance dysfunction, epithelial barrier abnormalities, and a dysregulated immune response. For the classification of secondary chronic inflammation of the paranasal sinuses, a division was introduced that is also dependent on the anatomical location and the dominant endotype to which it belongs: mechanisms inducing the inflammatory process, mechanical changes, local changes, and the phenotypes included in Figure 1B.

## 3. The Natural Microbiota of the Sinuses

Similarly to the body’s other microbial niches, the nasal microbiome develops throughout a person’s life. The fetus resides in a sterile uterus before birth. The newborn’s first contact with microorganisms from the vaginal canal occurs during natural childbirth. Performing a cesarean section allows the newborn to come into contact with the skin. The microflora of the nose and nasopharynx start forming after birth. Several factors play an important role in the formation of the early microbiome, including breastfeeding. Still, the diversity of the microbiota of newborns at birth remains low. This bacterial diversity increases during the first few months until age three. Then, the bacteria in the upper respiratory tract become more stable, and the microbiome of children becomes similar to that of adults.

The nasal cavity contains a diverse microbial community. The healthy nasal cavity is colonized by strains of the *Corynebacterium*, *Staphylococcus*, *Streptococcus*, *Dolosigranulum*, and *Moraxella* genera [14]. The deeper areas of the nasal cavity and sinuses have unique local microenvironments (pO2, pH, etc.) and immune properties. Examining the sinus microbiome in healthy individuals is difficult, mainly due to the invasiveness of the test in the absence of clinical indications to collect specimens [15]. Reduced bacterial species richness and diversity are often found in CRS [16].

Many factors have been proven to affect the sinus microbiome. In addition to significant inter-individual variability, age, and smoking, the composition and distribution of individual microorganisms are also affected by the frequency of antimicrobial use. These agents disrupt the balance of the microbiome, leading to microbial selection and possible superinfections caused by more than one pathogen [17].

The presence of live bacteria in healthy sinuses is well documented. It should be noted that the total number of bacteria present in healthy and diseased sinuses appears to be the same [17]. Pathogenic organisms are usually found in small numbers in healthy sinuses and can be a temporary or permanent component of a healthy sinus microbiome [15]. High inter-individual variability of the microbiome is often observed in healthy controls and patients with CRS. Disruption of the stable microflora can contribute to the exacerbation of chronic inflammatory disease or the onset of acute infection. As a result of dysbiosis, communities of “benign” microorganisms become pro-inflammatory, invasive, or allow excessive growth of pathogenic microorganisms. There is also growing evidence that dysbiosis of the sinus microbiota has been linked to the pathogenesis of CRS. Human studies have shown that the CRS microbiome is characterized by a loss of diversity compared to healthy controls, as indicated by an intense increase in specific microbial populations [15].

There is currently no consensus on the most common bacteria found in healthy and sick persons. There is no clear answer as to which microorganism is “causal” and which is “protective”. Bacterial communities vary widely, probably due to the heterogeneous nature of the disease and different patient populations, but also due to differences in sampling techniques, choice of bacterial primers, sequencing methods, and data analysis. Comparing the studies is extremely difficult. Nevertheless, several patterns are emerging. Bacteria belonging to the species *Propionibacterium acnes*, *Staphylococcus epidermidis*, and *Staphylococcus aureus* and the genus *Corynebacterium* spp. were often identified as the dominant species in healthy subject groups [18]. Organisms such as *S. aureus* and coagulase-negative *Staphylococci* can behave as commensal strains or as pathogens, depending on gene expression, environmental conditions, and interactions with other microorganisms [18]. At low concentrations, *S. aureus* can induce the anti-inflammatory cytokine IL-10, but high concentrations were found to promote IL-10 reduction, leading to a pro-inflammatory response [15]. Strains of *S. epidermidis* directly produced a serine protease that inhibited *S. aureus* biofilm formation. At the same time, strains of the *Corynebacterium pseudodiphtheriticum* species are characterized by opportunistic activity against *S. aureus* strains [18]. Commonly identified strains of *P. acnes* in healthy mucosa have been shown to produce bacteriocin, which acts as an antimicrobial and antifungal compound. In this way, they can modulate the immune response against pathogenic bacteria [15].

## 4. Etiological Agents of Sinusitis

Numerous hypotheses regarding the pathogenesis of CRS have been described in the literature. Researchers have mentioned that etiology may be related to superantigens, bacterial biofilm, microbiome, the presence of fungi, the action of eicosanoids, and the functioning of the immune system [19].

Viral CRS is characterized by a tendency toward self-limitation, and treatment is symptomatic (analgesics, antipyretics, and/or medications to reduce congestion). Clinicians assume that sinusitis can be attributed to a viral etiology if symptoms are present for less than ten days and the patient’s condition does not deteriorate. If symptoms persist or worsen, acute bacterial sinusitis is then suspected, possibly developing into chronic rhinosinusitis [20]. Most cases of bacterial sinusitis are a continuation of untreated viral sinusitis. Chronic sinusitis is also favored by nasal polyps, deviated nasal septum, facial trauma, respiratory infections, allergies, and other diseases, such as cystic fibrosis, gastroesophageal reflux, HIV, immune diseases, and exposure to environmental pollution [21,22]. Most of the aforementioned factors and conditions that predispose to recurrence show a strong connection to the immune system. In addition, through their location, the maxillary sinuses are prone to complications from poorly selected dental treatment, including implant treatment [9].

The relationship between allergies and chronic sinusitis has been widely researched. Some reports in the literature support a cause-and-effect relationship between allergy and chronic sinusitis, while others do not confirm such an association. A representative summary is found in Wilson’s work on this subject, published in 2014 [10]. The author reviewed 24 studies, 18 of which concerned the relationship between allergy and chronic rhinosinusitis without nasal polyps. Eleven studies indicated a relationship between both diseases, and seven showed no correlation. Wilson also assessed nine studies that investigated the relationship between allergy and CRS with the presence of polyps; four studies showed an association between allergy and CRS with the presence of polyps, and five studies showed no relationship. Therefore, an almost equal number of studies confirmed and denied the association of allergy with CRS both in the presence of polyps and without polyp [10]. At the same time, it was confirmed that specific subtypes of CRS, such as allergic fungal rhinosinusitis and central compartment atopic disease, have a stronger relationship with allergy than other subtypes [10].

The relative lack of research on fungi and viruses, when compared to bacterial research is a result of delay in research of microbiome. There has been a focus on microbial detection techniques, mainly on numerically dominant bacteria [15]. However, viral replication can cause epithelial damage and increase bacterial adhesion to the mucosa, while fungi can act synergistically with pathogenic bacteria, playing a role in the pathogenesis of CRS. The exact role of these organisms in the pathogenesis of CRS and the etiological significance remain poorly understood [23].

The presence of anaerobic microorganisms has been observed in studies on CRS, which can be explained by the selective pressure of antimicrobial agents that allow the growth of anaerobic organisms and the presence of conditions suitable for growth (i.e., sinus hypoxia). Many studies on CRS have reported that anaerobic microorganisms such as *Peptoniphilus*, *Anaerococcus*, and *Prevotella* occur in patients with CRS. Environmental conditions in the sinus cavities are not a cause of hypoxia, especially after opening the sinuses during endoscopic sinus surgery. The expansion of anaerobic microorganisms is due to local conditions present in the mucus or bacterial biofilms. Oxygen levels in sinus mucus are dynamic and dependent on host and microbial influences [23].

### 4.1. CRS as a Social Disease

Sinusitis is one of the most common infectious conditions in developed countries, being reported in 10 to 20% of patients [9]. CRS is a debilitating disease that negatively affects quality of life and poses an economic burden on society [14].

CRS is defined clinically based on the presence of symptoms that persist for more than three months, which include positive endoscopic and/or CT scan results. CRS can be further classified based on the presence of nasal polyps, such as CRS with nasal polyps and CRS without nasal polyps. Clinically, it is important to distinguish between these forms of CRS, as patients in the first group have a higher burden in terms of disease severity and poor treatment outcomes compared with those in the second group [24]. Despite advances in surgical methods and treatment, little improvement has been seen over the past two decades, and it is estimated that symptoms do not resolve with therapy in 30% of those suffering from CRS. Moreover, a classification system based solely on dividing patients with CRS into a group with nasal polyps and a group without coexisting nasal polyps is outdated and provides a very limited picture of the heterogeneous pathophysiology responsible for CRS symptoms. A redesign of current guidelines is needed, with an emphasis on characteristics that can be measured and treated, such as the type of inflammation present in the sinuses. Stratification according to the degree of eosinophilia and/or neutrophilia in the tissue would provide better guidance for the clinician [25].

CRS in children and adults differs due to anatomical and histopathological differences and the maturity of the immune system. Predisposing factors, such as frequent viral infections of the upper respiratory tract and enlarged tonsils in the pediatric population, are also important. The main symptom observed in children is a persistent cough, and therapy is primarily based on pharmacological treatment. A lack of clinical improvement allows for the consideration of surgical treatment [26].

In the elderly, the nasal mucosa is thinner, which may be due to reduced blood flow to the nasal cavity and reduced mucus production. Changes in the epithelium associated with age may contribute to weaker pathogen removal, dysbiosis of the nasal microbiome, and consequent transfer of oral and pharyngeal microflora to the nasal cavity. In addition to physiological changes, the immune response also changes in older people. An impaired immune response to new and existing pathogens in the elderly may be an important factor in the greater susceptibility to infection, chronicity of CRS, and possibly the development of other inflammatory diseases in this population [14].

Any abnormal activation or lack of immune system suppression can lead to chronic inflammation. Since bacterial pathogens are frequently observed in CRS, it has been speculated that bacterial dysbiosis plays an important role in initiating or contributing to CRS [14]. Dysbiosis of the microflora can be associated with various diseases, including asthma, allergic rhinitis, atopic dermatitis, cardiovascular disease, obesity, diabetes, and neurodegenerative diseases [27]. Due to the proximity of the nasal cavity to the brain, inflammatory diseases of the nasal cavity, such as acute and chronic rhinosinusitis, can initiate a number of neurological complications, including epidural abscesses, meningitis, cerebral abscess, cerebral venous sinus thrombosis, and orbital cellulitis. These infections have common consequences, such as permanent vision changes and epilepsy. Additionally, diagnosing and treating the listed conditions comes with a heavy financial cost burden [14].

### 4.2. Changing Sinus Microbiota in Chronic Conditions—Current State of Knowledge

CRS is characterized by a significant disruption of the resident microbial community, with reduced richness, changes in composition and a distorted abundance of native microorganisms. Views on the pathogenesis of CRS have evolved beyond a disease state arising from infection with specific pathogens into a complex condition associated with a disruption of the underlying microbiome. Microorganisms interact with each other and their hosts in mutual and antagonistic ways. These interactions include nutrient ingestion, secretion of antimicrobial agents, and competition at adherence sites.

The presence of pathogens on the surface of the sinus mucosa does not confirm an infection. Opportunistic pathogens do not pose a threat as long as they occur in small numbers. The disruption of the core microbiome (i.e., dysbiosis) may be a critical prerequisite for disease development. A disturbance of immune homeostasis creates favorable conditions for the development of CRS, where the proportion of commensal and pathogenic bacteria is disturbed in favor of pathogenic bacteria [28].

Innate immunity, acquired immunity, mucosal integrity, wound healing, and other host-microbe interactions can be disrupted by microbial imbalances. Thus, the resident microbiome may not only be crucial for pathogen exclusion but also serve as a disease modifier through its regulatory influence on the host immune system. As such, maintaining homeostasis of the resident microbial community may be crucial to maintaining overall health and preventing sinus infections [28].

The type and amount of microflora in the mucosa of patients with CRS changes significantly. Changes in the microbiota are linked to various factors. In addition to significant differences between individuals, age and smoking also affect the composition and distribution of microbial species. The typical microbiota in patients with CRS also varies by geographic location [5].

As with many other chronic inflammatory diseases, a breakdown of the local microbiome, characterized by the expansion of pathogenic bacteria and degradation of the commensal bacterial populations, is characteristic of patients with CRS. Although there is a growing body of research on the microbiome in CRS, the exact role of microbes in the pathogenesis of CRS at the community and individual species levels remains poorly understood [26]. Commensal bacteria are key components in the development of mucosal barrier function and play an essential role in the innate and acquired immune response. They also inhibit the establishment of pathogens [27].

In the past, the study of bacteria in acute and chronic rhinosinusitis has mainly involved interactions between a single bacterial pathogen and its host. The development of laboratory methods in microbiology and the understanding of the sinus microbiome have contributed to an increased number of studies examining the microbial community in the biofilm as an inseparable, interconnected unit [4].

Most cases of sinusitis are viral, without the need for antibiotic therapy in their initial phase. However, CRS can also be caused by bacterial agents including *Streptococcus pneumoniae*, *S. aureus*, and coagulase-negative *Staphylococci*. as well as Gram-negative bacteria (*Haemophilus influenzae*, *Moraxella catarrhalis*, *Pseudomonas aeruginosa*, *Proteus* spp., *Klebsiella* spp., *Enterobacter* spp., and *Escherichia coli*) and anaerobic bacteria (*Peptostreptococcus*, *Prevotella*, *Porphyromonas*, *Bacteroides*, and *Fusobacterium* spp.) [29,30,31]. Fungi from the Mucoraceae and Trichocomaenae families may be responsible for sinusitis in immunocompromised patients [32].

As in the intestines, changes in the homeostasis of the nasal and sinus microbiome can play a significant role in the progression of diseases such as CRS, allergic rhinitis, and asthma. Bacterial dysbiosis can start in early infancy or develop later in life. For example, Teo et al. observed nasopharyngeal bacteria in infants during their first year of life; they showed that a specific bacterial composition in the nasopharynx is a predictor of future asthma development in these infants, with strains belonging to the *Streptococcus* genus being the main contributor to this result [33]. The study highlighted the importance of the nasal microbiome composition in infants as an indicator of future chronic inflammatory lung disease. Another case–control study highlighted the role of the nasal microbiota in early life in the development of upper respiratory allergies in infants. In healthy children, the diversity of nasal microbiota increases with age, while diversity decreases with age in children with rhinitis. Thus, the nasal microbiome may play an important role in the development of airway inflammatory diseases [14].

A study conducted by Park et al. showed that, at the genus level, strains of the genera *Haemophilus* (26.8%), *Staphylococcus* (12.4%), *Bacteroides* (9.9%), and *Corynebacterium* (7.9%) predominated in the group of children and adolescents with CRS [26]. In the adult group, *Corynebacterium* strains were most abundant (25.1%), followed by *Staphylococcus* strains (13.1%). Comparing the two groups, the authors found that only *Corynebacterium* strains showed significantly higher relative abundance in the adult group than in the pediatric group. In addition, at the species level, *H. influenzae* strains were most abundant among children and adolescents (22.0%). In the group of children and adolescents, *S. aureus* (11.9%), the genus *Corynebacterium* (7.6%), *Bacteroides vulgatus* (7.1%), and *S. pneumoniae* (6.8%) predominated. In adults, *Corynebacterium* spp. were most abundant, such as *Corynebacterium accolens* and *Corynebacterium tuberculostearicum* (23.7%), followed by *S. aureus* (8.9%) [26].

A study presented by Psaltis et al. showed that CRS samples were less diverse than healthy samples and were significantly enriched with the presence of bacterial virulence genes and the production of antimicrobial metabolites [29]. Further studies showed that CRS samples were also enriched with mucin-degrading genes. *Prevotella*, *Fusobacterium*, and *Streptococcus* were among the strains responsible for mucin degradation. Degradation of mucin by these genera enhanced the growth of *S. aureus* strains [29]. In other studies [30,31], *E. coli* strains associated with virulence factors (*fimG/H*, *fyuA*, *agn43*, *hlyA*, *usp*) were detected in intraoperative biopsies. The authors suggested that the expression of three genes, *fimG/H*, *fyuA*, and *agn43*, could lead to the formation of a “super” biofilm in CRS.

The composition of the sinus microbiota can be affected by the performance of a surgical procedure [26]. The number and diversity of fungi in the sinus cavity of patients after endoscopic sinus surgery is significantly reduced. The results of a meta-analysis showed that bacterial richness and diversity in CRSs decreased after surgery. This means that certain pathogenic microflora, which are usually present in small numbers, can dominate the microbiome under disease conditions. Disruption of microbial communities leads to the loss of key symbiotic species [5].

## 5. From Classical Microbiology to Molecular and OMICS Methods

Differences in the structure of the sinus microbiome may be due to differences in the collection sites (sinuses, middle nasal meatus), the instruments or techniques used to collect the sample, and the type of specimen collected [17].

Sampling techniques and sample types vary from study to study, ranging from swabs and/or brush swabs, swabs taken under endoscopic guidance, mucosal biopsies, and material taken during nasal and sinus rinses. Samples of the sinonasal microbiome can be problematic to process and analyze due to low microbial biomass and high contamination of host DNA. Studies of multiple sampling sites in a single patient showed variability in the composition of the sinus microbiome, but overall, interpersonal variability far outweighed intrapersonal variability [29].

Samples taken from the nasal auricles are unlikely to be representative of the sinus microbiome. Differences in sampling techniques can significantly affect the results, including the detection of anaerobic bacteria, the abundance of which can vary in different locations of the sinonasal cavity. Although surgically collected sinus tissue is the most representative material for study, collecting a large series of specimens from patients with CRS and healthy controls is difficult and would limit the data to mainly patients requiring surgical intervention [16].

The microbiome of the anterior part of the nasal cavity appears to differ from the microbiome of the deeper parts, such as the middle nasal meatus, where the openings of the frontal sinus and maxillary sinus are located, and from the microbiome of the sphenoethmoidal recess. The middle nasal meatus is often considered a representative sampling site. Because of its relative ease of access in the clinical setting and its presumed similarity to the deeper sinuses, this area has been used for sampling in numerous tests to date [15]. A study involving 225 CRS patients and 100 controls showed that the middle nasal meatus could represent the microbiome of the sinus, confirming the results of previous smaller-scale tests comparing the composition of the microbiome taken from the middle nasal meatus with that of the maxillary and ethmoid sinuses. These findings support the feasibility of sampling more accessible sinonasal sites (such as the middle nasal meatuses) during out-of-hospital or clinic visits [30].

The key to microbiological testing is proper specimen collection. Sampling by traditional methods involves the risk of contamination due to the presence of randomly collected microorganisms. The antibiotic therapy then used is based on identified incidental microorganisms, diverting attention from the actual causative agent of the disease. The use of an invention, with an application filed to the Patent Office by the author [34], allows for the non-invasive and rapid collection of diagnostically relevant samples for microbiological testing. The invention consists of a set of cannulas intended for collecting microbiological samples of highly diagnostic specimen from diseased paranasal sinuses (Figure S1).

The dedicated tip allows precise access to natural sinus openings or sinus openings created during surgery, avoiding contact with other tissues. Samples obtained by using the invention reflect the microbiological status of the sinuses. A smear from the surface of the sinus mucosa is an improperly collected material that can misdirect therapy. The use of an advanced angular model of the sinus mouth effectively reduces such errors. In addition, it allows precise collection of material for examination without artifacts or contamination by microorganisms present in other structures outside the sinuses. The use of targeted antibiotic therapy based on the antibiogram result from the next step eliminates bacteria that invade mucous membranes, intercellular spaces, or deep tissues [34]. The use of the angled cannula during the procedure is shown in Figure S2.

The ability to accurately collect specimens for testing may allow for more effective treatment regimens and provide a better basis for clinical and laboratory studies of CRS [31]. Bacteriological studies in chronic rhinosinusitis are widely conducted using culture techniques. Traditional culture-dependent techniques involve sampling the surface of the nasal mucosa and then growing the microorganisms on the surface of the medium or inside the medium. These techniques often fail to cover the entire diversity of microorganisms in a sample, as the culture medium may not provide the conditions required for the growth of many microorganisms. Traditional techniques are still cheaper and allow in vitro determination of pathogen susceptibility to antibiotics. Newer culture-independent molecular methods include immunological, nucleic acid-based, gene-targeting, or meta-omics techniques. Immunological techniques include ELISA, serological tests, and microarrays. These tests have a moderate level of sensitivity and a moderate level of specificity and have the advantage of being quick and relatively inexpensive. Disadvantages include limited detection of microorganisms present in small numbers and technical difficulties in producing highly selective antibodies.

### 5.1. Contemporary Research Trends in CRS Diagnostics

According to WHO criteria, a patient history is necessary to confirm nasal blockage and an imaging test is needed to confirm nasal mucosal edema. The scientific research being conducted is increasingly based on the use of simpler and more objective analytical methods. They may, in the future, be the primary method of diagnosing or a screening test for sinus diseases. A fairly general screening test for sinusitis is the determination of airway nitric oxide (II), a biomarker of airway inflammation. Measuring the concentration of this compound can also be helpful in diagnosing variant CRS [35]. However, we often see contemporary research trends in molecular diagnostics.

#### 5.1.1. Sensors

Bacterial assaying involving a sensor system using metalloporphyrin dyes as sensors is an example of a colorimetric method [36]. Exhaled air samples were analyzed to distinguish CRS patients from healthy ones. Gas taken from the nasal cavity was dropped onto a matrix with 36 sensors, and then the color changes in the sensors were recorded on an RGB scale. This method was 90% effective in identifying patients with chronic rhinosinusitis. A major advantage of this method is the ease of finished device use, as it does not require specialized knowledge, and the key element is correct sampling.

Exhaled air samples can also be analyzed by methods that use modified nanostructures such as gold nanoparticles or carbon nanotubes. The effectiveness of this method in detecting chronic rhinosinusitis was 85%. It is also useful in identifying types of CRS [37].

It is also worth mentioning a hybrid sensor for the detection of methicillin-resistant *Staphylococcus* strains (NanoLantern-TM strain) [38]. It is based on a fluorescent-labeled DNA fragment and is placed on gold so that the signal can be processed. Optical biosensors based on chemiluminescence have also been used. *S. aureus* cells were labeled with horseradish peroxidase, retained on a membrane, and detected by a luminometer [39].

#### 5.1.2. Nucleic Acid-Based Tests

##### Microbiota Detection

Nucleic acid-based tests such as PCR have excellent specificity and the advantage of providing the most detailed, unbiased information, which provides the opportunity to identify new microorganisms. The test material can include swab samples, mucosal tissue, or intra-operative liquid biopsy. Samples taken from sinus mucosal tissue and sinus swabs were compared. No significant differences were detected in the microbiota of the samples, suggesting that the use of less invasive swabs is warranted [40].

A molecular technique designed to analyze the microbial structure of the environment is based on PCR and restriction fragment analysis using fluorescently labeled primers (terminal Restriction Fragment Length Polymorphism (t-RFLP) [41]. The selection of a suitable universal genetic marker, common to the entire population of studied microorganisms, i.e., specific to bacteria or a particular taxonomic unit (family or genus), combined with restriction enzyme digestion, yields a characteristic pattern for a given microbial community. The 16S rRNA gene is often used in this method. Fluorescently labeled primers allow for the production of amplicons with an integrated fluorophore group from the 5′ end. The appropriate selection of a restriction enzyme (the recognition site for endonucleases should be at different distances from the primer hybridization site) results in characteristic digestion product length profiles for individual microorganisms. Using the t-RFLP method, a simplified formula is obtained to assess both phylogenetic diversity and community composition. Nevertheless, the use of universal or domain-specific primers allows very general phylogenetic conclusions to be drawn. The use of phylogenetic group-specific primers helps increase the degree of species identification for microorganisms. This approach allows for the analysis of the diversity of microorganisms and the assessment of changes in the structure of the microbial community over a defined period of time and space that can occur in response to factors that disrupt their natural environment. t-RFLP is an inexpensive method relative to sequencing, characterized by reproducibility and high throughput. It is also suitable for studying dynamic changes in complex microbial ecosystems over time, including the characterization of the diverse bacterial community in CRS [42]. Stressmann et al. characterized 70 clinical samples from 43 CRS patients undergoing endoscopic sinus surgery by using this technique [39]. Distinctive band patterns were obtained and assigned to 34 genera after cloning and sequencing. The predominant species were those belonging to the genera *Pseudomonas*, *Citrobacter*, *Haemophilus*, *Propionibacterium*, *Staphylococcus*, and *Streptococcus. Pseudomonas aeruginosa* was the most common species [42].

In the era of sequencing methods, two approaches to analyzing genetic material can be distinguished. The first is sequencing whole genomic DNA from the tested environment (shotgun metagenomics) [43]. This method is useful for detecting the presence of strictly defined species in a sample. It also facilitates the testing of viruses, which are difficult to detect due to the wide variety in their genetic material. The second and currently more common approach is sequence analysis of amplified specific genes, or the so-called marker genes (Sanger method, Illumina Hi-Sec, Illumina Mi-Seq) [43]. Sequencing of the gene encoding the 16S subunit of ribosomal RNA (16S rDNA) is often used in bacterial identification. It is an evolutionarily conserved gene about 1500 nucleotides long, encoding a fragment of the small subunit of ribosomal rRNA. The single-gene target approach based on 16S rDNA is the gold standard in microbial typing. Most 16S rRNA-based genotyping protocols use V5-V6, V3-V4, or V4 hypervariable regions to identify and catalog microbial profiles. This approach provides information on species richness, evenness, and dominance (alpha diversity); differences among microbial communities from different patient groups; and differences among samples within a sample type group (beta diversity). Currently, it is recommended to sequence the amplicon of the hypervariable region of the 18S rRNA gene (18S rDNA) or the internal transcribed region in the case of fungi. Hauser et al. [44] showed that the detection of bacteria using 16S rRNA gene sequencing allowed for greater sensitivity and provided more information on bacterial diversity than standard cultures. In another study of the composition of the bacterial community of the microbiota based on 16S rRNA gene sequences using Illumina MiSeq, members of the genera *Streptococcus*, *Haemophilus*, and *Veillonella* were found to be strongly correlated with CRS [45].

##### Human Factors and Susceptibility to CRF

CRS has been the subject of multiple genetic susceptibility studies. The phenotypic classification of CRS was mainly based on the presence (CRSwNP) or absence of nasal polyposis (CRSsNP); thus, genetic studies are mostly related to this division. Distinct clinical phenotypes associated with aspirin-exacerbated respiratory disease (AERD), allergic rhinosinusitis, and systemic diseases such as cystic fibrosis (CF) and autoimmune/vasculitis have also been identified. Current studies suggest that the inflammation of CRS varies widely, with three main inflammation endotypes: T1 with elevation of T1 cytokine IFN-γ, T2 with eosinophilia and elevation of T2 cytokines, and T3 with neutrophilia and elevation of T3 cytokines including IL-17A [46].

A recent investigation by Stevens et al. has clarified the associations between endotypes and clinical presentations [47]. The T2 endotype was associated with loss of smell, asthma comorbidities, and nasal polyposis, whereas the T3 endotype was associated with the presence of intraoperative pus, indicating an association with infection. Associations of endotypes in CRSsNP and CRSwNP were discovered. The authors found that odor loss was still associated with the T2 type and pus with the T3 type in both CRSsNP and CRSsNP. CRS patients with mixed T2 and T3 endotypes were more likely to have clinical presentations shared by both T2 and T3 endotypes.

Genetic research can be used to analyze known genes for variability (frequency of SNP polymorphisms in selected alleles compared to controls) or genome-wide association studies (GWAS) to identify novel SNPs [48].

*CRS and polymorphism genes in relation to inflammation reaction and innate immunity*

Cytokine levels expressed by TH-2 cells in CRS depend on the patient subgroup. Polymorphic variants rs1881457 and rs1800925 of the *IL-13* gene were detected only in patients with aspirin-dependent asthma. Polymorphism of the *IL33* gene, which codes for interleukin-33 with a cytokine response function for the production of Th2 cytokines [49], and *IL1RL1*—interleukin-1 receptor-like1 gene (receptor for IL-33)—which acts as an effector molecule of the Th2 response, are among the genes having a significant association with asthma and CRS [50].

Whereas IL22RA1 (interleukin-22 receptor, subunit alpha 1), with cytokine receptor functions, mediates innate immune response and is characterized by a decreased level of expression gene in patients with recurrent CRSwNP [51], *Il-4* (cytokine, Th2 response) and *IL-6* (cytokine, production of inflammation) polymorphisms appear to also be associated with CRSwNP pathogenesis.

Another gene, *TGFB1* (transforming growth factor beta-1), which has cytokine functions and controls the proliferation and differentiation of many cell types, showed polymorphism in promotor regions, especially for rhinosinusitis in aspirin-intolerant asthmatic patients [52]. The *IRAK4* gene—interleukin-1 receptor-associated kinase 4—is responsible for downstream signaling of Toll-like receptors, together with the *TLR2* gene, and its polymorphism was found to correlate with CRSwNP patients.

Met proto-oncogene (*MET*) encodes a tyrosine kinase receptor, together with *PPP1R9B* (encoding protein phosphatase 1, regulatory subunit 9B), have increased expression in CRS [53]. This study was carried out to investigate CRS and aspirin-sensitive asthma.

Finally, another study found that the eosinophilic form of CRSwNP was statistically significantly associated with polymorphisms in the nitric oxide synthase gene *NOS2A* [54].

*The human leukocyte antigen (HLA)*

The HLA antigen complex, which functions as MHC class I or II receptors, has a significant role in disease and immune defense. HLA alleles such as HLA-A_24, HLA-A_74, HLA-B_54, HLA-B1_3, HLA-B1_08, HLA-B_07, HLA-B_57, HLA-Cw_04, HLA-Cw_12, HLA-DRB1_03, HLA_DRB1_04, and HLA-DQB1_03 have been reported in the literature to be associated mainly with CRSwNP and very often with different types of asthma [55,56]. HLA patterns were found to change depending on the endotypes and composition of a particular population.

*Genes responsible for tissue remodeling*

Tumor necrosis factor (TNF) is a pleiotropic cytokine produced by various cell types (activated macrophages, monocytes, and lymphocytes) and involved in a variety of pathological processes [57]. Elevated serum TNF-α levels have been observed in all endotypes of CRSwNP [58].

In CRS, the *TNF* gene (*TNFA*, *TNFAIP3*, *TNFB*) was found in several polymorphic variants, especially in the promotor region [59]. This polymorphism was associated with asthma and/or nasal polyposis, but the results were not unambiguous. According to Erbek et al., *TNFA* genotypes with a SNP (-238 AA and -308 GA) were associated with susceptibility to CRSwNP [60]. Szabo et al. reported that the *TNFA*-308 G>A polymorphism is a predisposition factor for CRSwNP in aspirin-sensitive Hungarian subjects [61]. Zhang et al. suggested that, in patients with significantly elevated TNF-α levels, an inhibitor TNF-α—etanercept—could be considered as a treatment option for CRS [62].

The other genes responsible for tissue remodeling that were analyzed in terms of variability and expression are the mucin (*MUC*) genes. MUCs are heavily glycosylated, high molecular weight glycoproteins with various expression profiles depending on the form of disease. Ali et al. examined *MUC* gene expression in nasal polyps and reported that expression patterns were highly variable between polyps [63]. The submucosal glands showed the most significant change in gene expression in nasal polyps. In both submucosal glands and epithelial cells of nasal polyps, MUC4 and MUC5AC were found to be important components. The other authors, Liu et al. (2020) [64] suggested that the expression levels of *MUC5AC*, *MUC5B*, and *MUC2* were significantly negatively correlated with the recurrence rate of nasal polyps [64]. Meanwhile, the MUC19 gene with rs2933346 and rs1492313 polymorphisms was associated with bronchial asthma [65].

*Genes encoding xenobiotic-metabolizing enzymes*

Xenobiotic-metabolizing enzymes that mediate both activation and detoxification must also be considered in the study of CRS. When metabolic activation exceeds detoxification, protein or DNA binding can occur, resulting in cytotoxicity, DNA damage, or other toxic effects. *GSTM1*, *GSTT1*, and *GSTP1* genes, encoding glutathione S-transferases, belong to the GST family enzyme and are important in xenobiotic-induced damage of the nasal mucosa [66]. Ozcan et al. reported a *GSTT1* gene polymorphism for non-allergic nasal polyposis and suggested its importance in the pathogenesis of NP [67]. On the basis of other research, both *GSTM1/GSTT1* null genotypes have been considered to be risk factors for the development of NP and hyposmia (reduced ability to sense odors) in allergic individuals [68]. The authors thus confirmed the *GSTT1* gene polymorphism as a prognostic marker for CRS.

*Taste receptors*

In a paper published in 2016, Polish researchers described their study of T2R receptors (specifically, T2R38s), which are specific regulators of the body’s immune function. Samples from sinus mucosal biopsies were analyzed. Their genotypes were studied using the Sanger method, and SNPs were identified. The *AVI/AVI* genotype was regarded as a non-functional variant for alanine, valine, and isoleucine. The expression of the *TAS2R38* gene in the collected mucosa was also studied. It turned out that CRS patients had high expression levels of this gene. It was concluded that certain variants of the *TAS2R38* gene may predispose to CRS and that the studied receptor and gene may constitute therapeutic targets for CRS treatment [69].

Another example of the practical use of the Sanger method in the diagnosis of CRS was described in a paper presenting the results of research on the *PARS2* gene, which encodes an enzyme catalyzing the ligation of proline to tRNA molecules. The low-frequency variant has been shown to be more common in CRS patients than in the control population. Further studies are anticipated due to the yet unknown role of this gene in the development of CRS [52].

*Association between mutation in the CFTR gene and CRS*

A genomic study conducted by Wang et al. [70,71] on material collected from patients with CRS confirmed the association observed between the occurrence of CRS symptoms and the presence of mutations in the *CFTR* gene, which affects impaired mucociliary transport. Pinto et al. found that the region of chromosome 7q31.1–7q32.1 that is associated with the *CFTR* gene may have a role in the development of CRS [72]. Young et al., based on reports from the USA and Europe, indicated that the prevalence of CFTR mutations in patients with CRS was 5.65% and that the prevalence of the Phe508 mutation was 4.22% [73]. According to recently reported data, *CFTR* polymorphism (e.g., M470V) [70] and G551D-*CFTR* mutation can predispose persons to CRS [74], especially CRSwNP. Gene target therapy has been introduced to treat G551D mutations [74].

*Genes in arachidonic acid metabolism associated with CRS*

Al-Shemari et al. showed that three SNPs that are located within the *ALOX5* (arachidonate 5-lipoxygenase), *CYSLTR1* (cysteinyl leukotriene receptor 1), and *ALOX5AP* (arachidonate 5-lipoxygenase-activating protein) genes belonging to the lipoxygenase (*LO*) pathway of arachidonic acid metabolism are associated with [75] CRS. In another study, De Alarcon et al. indicated that polymorphism of the *LTC4S* gene encoding leukotriene C4 synthase was associated with CRS but together with aspirin-dependent asthma [76]. The relationship between *PTGDR* (prostaglandin D2 receptor gene; it has a role in asthma and allergic diseases) and *LCT4* gene polymorphism was further demonstrated by Pescador et al. with a restricted statistical significance [76] for CRSwNP. The *COX2* variant rs20417 was also found in a population of Polish people with CRSwNP [77].

These relationships must be confirmed in extensive, well-designed studies.

Many genes were documented to have a genetic relationship with CRS, but most of them do not translate into replicability, depending on the variety of a population and the scale of the study group. Levchenco et al., based on the literature, assigned human populations from different continents to genes relating to CRS and their variability [65]. Genetic variability for SNPs was reported in European populations for the *TP73* (Tumor Protein P73) gene (rs3765731) and *IL1RL1* (Interleukin 1 Receptor-like 1) (rs13431828) [78]; *IL1A* (Interleukin 1 Alpha) (rs17561), *CD8A* (T-Cell Surface Glycoprotein CD8 Alpha Chain) (rs3810831), and *TAPBP* (TAP Binding Protein) (rs2282851) were detected in the Canadian population; and *B9D2* (B9 Domain Containing 2) (rs11466315) and *TLR2* (Toll Like Receptor 2) (rs3804099, rs3804100) were found in Korean populations. The American population was characterized by *IL1B* (Interleukin 1 Beta) with rs16944 as a frequent polymorphism [65].

**Figure 2:**
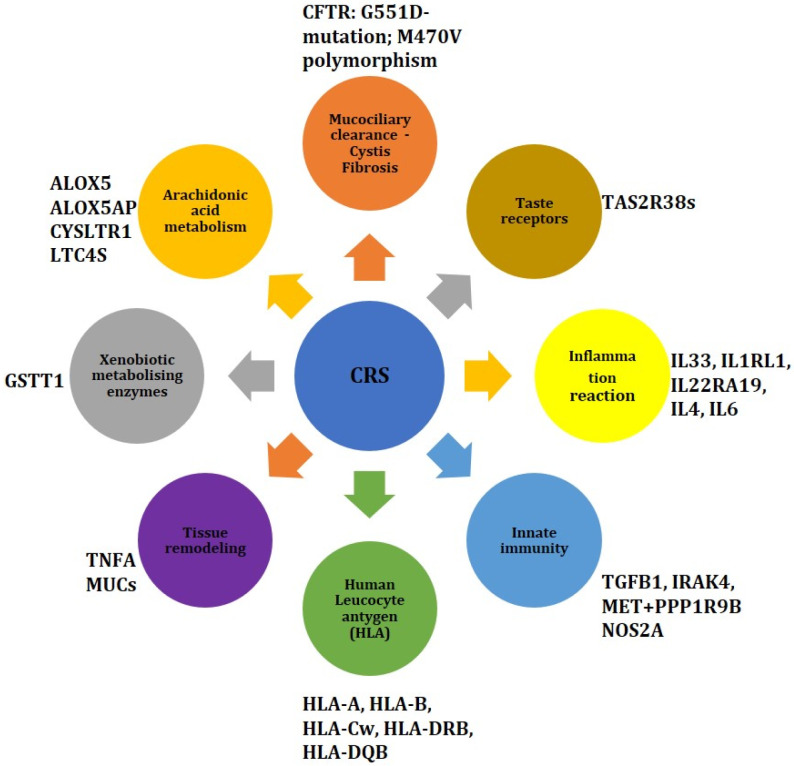
Summary of targets and genetic markers associated with CRS susceptibility. A description of the changes for particular genes is presented in the text.

The most documented genetic markers associated with CRS susceptibility are summarized in Figure 2.

##### Nucleic Acid-Based Test Problems

Sample transport, storage, and handling of clinical material to extract nucleic acids are very important in order to properly analyze CRS microbiota. Improper sampling risks contamination and the generation of false results [79]. Maintaining aseptic working conditions (gloves, masks, caps) prevents cross-contamination. It is recommended that the samples be transported placed on ice, without drastic changes in temperature [70]. Further storage of samples should take place at −80 °C. The sequencing of 16S rRNA amplicons has shown that even short-term storage of samples at higher temperatures can significantly alter the microbiome structure [80].

The first step after collecting the sample is to isolate the genetic specimen. This can be performed using off-the-shelf commercial reagent kits or appropriately selected reagents. Various methodologies are known for the extraction of nucleic acids, including mechanical lysis using bead beating or chemical lysis with the supplementation of various enzymes, such as lysozyme, lysostaphin, mutanolysin, lyticase, and proteinase K. It is advisable to remove biological material other than microbiota; therefore, it is worth using saponins before extracting the nucleic acids of microorganisms, allowing selective lysis of human cells. Some of the microorganisms may be lost due to improper pretreatment of the biological material, and there may also be problems with sample contamination. The quantitative and qualitative differences in the microbiota and the structure of the bacterial cell wall can significantly affect the integrity of the bacterial profile. Checks on the efficiency of total DNA isolation are good practice and require additional experiments to ensure that all procedures were performed correctly and that none of the steps yielded false-positive or false-negative results.

Sequencing the 16S rRNA gene measures the total or relative abundance of bacterial DNA and does not distinguish between actively growing, dormant, or dead biomass. As with all assays, it is important to be aware of such errors and integrate new innovative techniques, for example, by separating active cells from extracellular DNA and inactive microbial subpopulations [35].

Table 1 shows the advantages and disadvantages of classical culture techniques and molecular techniques.

#### 5.1.3. Influence of Environmental Factors on CRS concerning Expression and Regulation of Genes

Non-infectious CRS may be caused by genetic factors, environmental factors, or both. Environmental factors can influence the function of genes. RNA sequencing (RNA-Seq), which is used for epigenetic studies, can investigate the effect of the environment on gene transcription. Variations in DNA methylation, histone modifications, and non-coding RNAs such as microRNAs (miRNAs) have been observed. These mechanisms can lead to the upregulation or silencing of genes. miRNAs can regulate human dendritic cell (DC) differentiation, maturation, antigen presentation, and cytokine profiles [81]. In a study by Ma et al. using RT-qPCR, it was shown that miR-150-5p was upregulated in DCs from different types of CRS patients [82]. According to the authors, MiR-150-5p regulated early growth response 2 (EGR2) and played an important role in the development of CRS.

Chronic sinusitis may be caused by exposure to air pollution. The Environmental Protection Agency (EPA) has defined six criteria air pollutants—particulate matter (PM_10_, PM_2.5_), ozone (O_3_), nitrogen dioxide (NO_2_), carbon monoxide (CO), lead (Pb), and sulfur dioxide (SO_2_) [83]. The severity of the disease and histopathological changes were also associated with increasing exposure to air pollution. Patients with CRS exposed to PM_10_ had impaired epithelial barrier function [84]. Chronic exposure to PM_2.5_ has also been associated with increased inflammatory changes and tissue remodeling in mouse models. Exposure to NO_2_ leads to an increase in inflammation and mucin production in the respiratory epithelium as a result of the production of reactive oxygen species. Transcription factor-related factor 2 (Nrf2) is a key regulator of oxidative and environmental stress. It has been reported that activation of Nrf2 by genetic or pharmacological approaches can attenuate the asthmatic phenotype in a mouse model of allergic asthma. The ability of Nrf2 activation to reverse PM-mediated sinonasal epithelial cell barrier dysfunction was reported by Nyall et al. [85].

Currently, CRS is more common in people who have smoked cigarettes. Drinking alcohol can also aggravate symptoms. In the CRSwNP group, significantly higher nasal levels of the eosinophilic biomarker ECP [86] were observed. Eosinophil development is regulated by a number of type 2 cytokines, primarily interleukin (IL)-5 [87]. Different endotypes are likely to have different eosinophil activation and recruitment drivers. In severe eosinophilic upper airway disease, nasal hyperreactivity to alcohol is significantly more common [86].

It may be possible to prevent disease onset or alleviate allergic symptoms by identifying specific environmental risk factors that interact with an individual’s genome. The discovery of more disease-causing genes could improve the diagnosis and treatment of allergic and CRS patients based on genetic profiling.

#### 5.1.4. OMICs Technologies

Analyzing the functionality of the microbial community using other OMIC technologies, such as genomics, transcriptomics, proteomics, or metabolomics, is crucial, as many microorganisms coexist in the sinus region with mutual relationships [15]. Genomics allows for the evaluation of the genome, transcriptomics evaluates mRNA, proteomics deals with the analysis of proteins, and metabolomics evaluates metabolites. By combining data from different OMICs technologies, it is possible to provide a more complete picture of complex molecular events and, most importantly, to understand the functioning of healthy and disease-altered cells. Analysis of the collected OMICs data opens up many new possibilities in treatment and diagnosis.

Proteomic studies allow the generation of protein profiles that can differentiate patients with CRS from control patients. Some of these proteins may be useful as potential biomarkers of CRS, such as the mucin glycoprotein. Mucus overproduction is responsible for the pathophysiological changes in CRS. Regulation of the expression of the mucins MUC1, MUC4, MUC5AC, and MUC5B is dependent on human neutrophil elastase (HNE), transforming growth factor-β1 (TGF-β1), and corticosteroids (CS) [88].

Other proteins that can be used as markers of inflammation in the nasal and paranasal sinuses are as follows: AMY1A—amylase α1A—a protein of glandular cell origin; calgranulin protein, expressed by macrophages in acutely and chronically inflamed tissues with bactericidal properties; and CCPBP (Clara Cell Phospholipid-Binding Protein), a protein of glandular cell origin that acts as a potent inhibitor of phospholipase A2 (an enzyme that plays a key role in initiating the metabolism of arachidonic acid to leukotrienes and prostaglandins) [89,90]. In CRS patients, all of these were found to be overexpressed. However, studies should be confirmed on a large population of patients with varying disease severity [32].

Metabolomic studies by flow-injection/electrospray ionization-tandem mass spectrometry (FI/ESI-MS/MS) confirmed the presence of several different classes of lipids. These included, among others, ceramides, phosphatidylethanolamines, phosphatidylcholines, and cholesteryl esters. Cholesteryl palmitoleate was present in patients with CRS and concomitant nasal polyps, whereas it was not detectable in patients with CRSsNPs [91]. Metabolomic studies have confirmed the importance of arachidonic acid metabolites in the pathogenesis of CRS, which correlates with the results of genetic studies. Neopterin is another metabolite that could be a potential biomarker for patients with CRS without polyps. Human monocytes and macrophages produce neopterin, and its amount determines the ability of these cells to produce reactive oxygen species. Significantly elevated neopterin levels were confirmed in CRS patients without polyps compared to healthy subjects.

**Figure 3:**
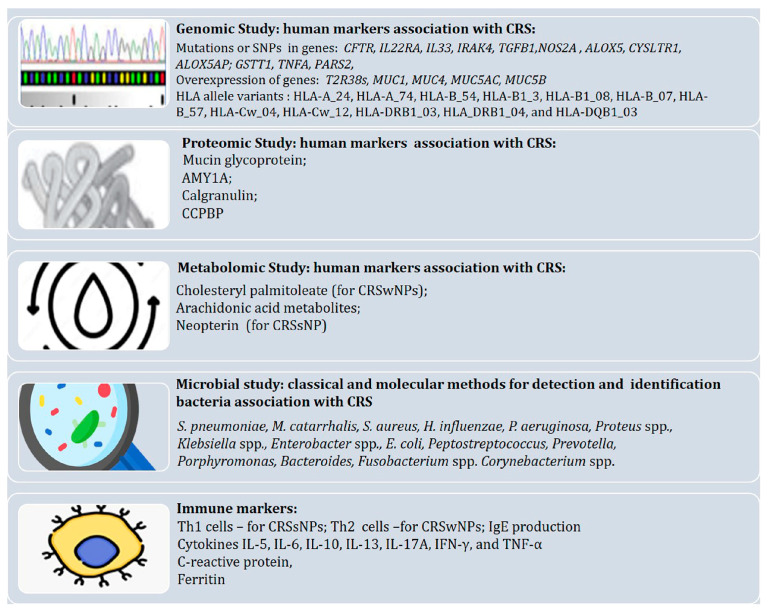
Promising markers for the diagnosis of CRS, obtained by genomic, proteomic and metabolomic analysis and the most commonly identified microbiota in CRS. Legend: ALOX5—arachidonate 5-lipoxygenase; ALOX5AP—arachidonate 5-lipoxygenase-activating protein; AMY-1A—amylase α1A; CFTR—cystic fibrosis transmembrane conductance regulator; CPBP—Clara cell phospholipid-binding protein; CRsNP—CRS without nasal polyps; CRwNP—CRS with nasal polyps; CYSLTR1—cysteinyl leukotriene receptor 1; GSTT1—glutathione S-transferases; HLA—human leukocyte antigen; IL22RA1—interleukin 22 receptor subunit alpha 1; IL-33—interleukin-33, IRAK4—interleukin-1 receptor-associated kinase 4; MUC1, MUC4, MUC5AC, MUC5B—mucins family; NOS2A—nitric oxide synthase gene; PARS-2—prolyl-tRNA synthetase 2; T2R38s—bitter taste receptors 38; TGFB1—transforming growth Factor Beta 1; Th1, Th2—type cytokines; TNFA—tumor necrosis factor gene.

A summary of the most important and promising markers in CRS and microbiota is shown in Figure 3.

## 6. The Problem in the Treatment of CRS—Antibiotic Resistance

The treatment of CRS includes using intranasal irrigations and topical and systemic pharmacotherapies, including corticosteroids, congestion-reducing drugs, antihistamines, antibacterial and antifungal drugs. Functional endoscopic sinus surgery or other ENT procedures are used in patients refractory to non-surgical treatment [20].

In clinical practice, there is a significant population of patients with chronic rhinosinusitis who remain resistant to treatment despite rigorous treatment regimens. Thus, personalized treatment based on the pharmacokinetic and pharmacodynamic properties of drugs is essential [9].

Guidelines differ as to whether antibiotics should be included in the treatment regimen for patients with CRS; this is due to insufficient evidence confirming their effectiveness and an incomplete understanding of the role of microorganisms in the pathogenesis of CRS [92].

According to the EPOS2020 guidelines, the treatment regimen for patients with chronic sinusitis includes intranasal glucocorticosteroids and the use of saline spray. Lack of improvement in health after six weeks is an indication to visit an otolaryngologist. In the next stages, it is recommended that the patient be treated based on a complete physical examination, taking into account comorbidities and a complete laryngological examination using endoscopic techniques. The detection of unilateral lesions during endoscopic examination is an indication for immediate imaging (preferably computed tomography or magnetic resonance imaging) due to the need to exclude cancerous lesions. Radiological confirmation of unilateral pathology is an indication for surgical treatment. The presence of bilateral, generalized lesions requires distinguishing whether it is a primary or secondary form of CRS and diagnostics to determine the phenotype of the disease, which directs further therapeutic procedures. New in EPOS2020 is the use of biological therapy in the treatment regimens of severe recurrent forms of CRS with nasal polyps and aspirin in selected groups [93].

Historically, antibiotics prescribed for the treatment of CRS were selected empirically because swabs were not routinely collected in clinical practice. This approach meant that the choice of antibiotic and the duration of its use varied significantly depending on the physician’s training and experience and the clinical setting. When selecting a therapeutic agent, costs and antibiotic resistance rates were also taken into account [92].

Between 2000 and 2015, global consumption of broad-spectrum antibiotics almost doubled. Additionally, in some countries, many antibiotics are sold over the counter. The Food and Drug Administration has approved numerous antibiotics for the treatment of acute or indefinite sinusitis. No antibiotic has a Food and Drug Administration indication for use in patients with CRS [92].

There is a lack of high-quality prospective studies confirming the validity of antibiotic therapy in CRS. Most analyses of CRS treatment with antibiotics for periods of less than three weeks focus on the treatment of acute CRS exacerbations [92]. The use of antibiotic therapy should be based on evidence of the effectiveness of antibiotics confirmed by studies conducted on homogeneous populations—e.g., patients who have or do not have nasal polyps, who have or have not undergone endoscopic sinus surgery, who have or have not performed standard therapies (e.g., saline irrigation, topical corticosteroids). Antibiotics should be selected according to the needs of patients and the results of microbiological tests, including antibiograms [94].

Numerous studies have been conducted on the effectiveness of antibiotic therapy in CRS depending on the site of drug administration. The concept of local use of antibiotics in the treatment of chronic sinusitis assumed that, after topical administration, high concentrations of antibiotics would reach the diseased areas of the sinuses. Local administration would enable the antibiotic to penetrate through the bacterial biofilm while protecting against the effects of systemic administration of antibiotics. Toxicity of topical antibiotics is rare but may be of concern, especially with topical aminoglycosides. Long-term exposure may potentially have adverse effects on hearing or kidney function [94].

The study presented by Barshak et al. demonstrated that the distribution of topical drugs into the interior of the sinuses is very limited, especially in the case of CRS with mucosal edema. Studies using neomycin, tobramycin, and bacitracin did not demonstrate the effectiveness of local administration of antibiotics. Antibiotics were administered in the form of an aerosol. Research into the use of topical antibiotics to affect bacterial biofilms is ongoing [94].

The effectiveness of oral antibiotics is greatest in exacerbations of infectious CRS, especially in patients with purulent exudate. Retrospective studies examining the concomitant use of oral antibiotics and steroids have shown a reduction in symptoms, improvement in the patient’s condition, and a reduced likelihood of requiring sinus surgery [95].

A survey of members of the American Rhinological Society found that 54% of clinicians routinely administer antibiotics but have no evidence to support this practice. The same study found that 62% of physicians routinely administer postoperative antibiotics, with 76% citing reducing the risk of postoperative infections as the reason.

There are no clinical trials whose results would justify the administration of systemic antibiotics immediately before sinus surgery. A randomized trial showed that short-term surgical outcomes did not improve with postoperative antibiotics [92].

A few studies have shown moderate symptom relief in CRS patients with and without nasal polyps after taking macrolide antibiotics for three to six months [92]. The observed improvement may be due to the anti-inflammatory properties of macrolides rather than their antimicrobial effects. There is no evidence that the combination of amoxicillin and clavulanate has direct anti-inflammatory properties in the sinuses and nose. Studies are needed to clearly determine the effectiveness of the drug in patients with CRS, taking into account the frequency of its prescription [92].

Excessive and unjustified use of antibiotics is the main cause of antibiotic resistance in bacterial strains. Antibiotic resistance is a huge public health threat that requires constant attention.

The Centers for Disease Control and Prevention estimates that more than 2 million patients in the United States suffer from complications related to antibiotic resistance, and 23,000 patients die from such complications each year. Moreover, the Centers for Disease Control and Prevention estimates that at least 1 out of 3 antibiotic prescriptions are unnecessary, and most unnecessary antibiotics are prescribed for respiratory diseases caused by viruses. Reducing the inappropriate use of antibiotics is key to combating antibiotic resistance [92].

Pathogens that cause chronic rhinosinusitis, especially Gram-positive bacteria, are becoming increasingly resistant to antibiotics, especially beta-lactams [23]. Methicillin-resistant or extended-spectrum β-lactamase-producing strains of *S. aureus* are becoming increasingly common in patients with CRS. In addition, antibiotic resistance affects the efficacy of cephalosporins of different generations in this patient group. The development of pathogenic bacterial resistance to many antimicrobial agents has become a threat to public health, as fewer and fewer antimicrobials are available to effectively treat these infections. The problem continues to grow, and precise definitions are needed to describe and classify multidrug-resistant bacteria so that reliable epidemiological data can be collected and compared between healthcare facilities in different countries [23].

Michalik et al. conducted a study of aspirates from the maxillary, frontal, and ethmoid sinuses of 380 patients with CRS [96]. The material was collected during the functional endoscopic sinus surgery (FESS) procedure. A total of 1232 strains were isolated from the study material. Among them, 580 were strains of coagulase-negative staphylococci, including 507 cases of *S. epidermidis*. The strains studied were resistant to different groups of antibiotics. The research showed that 20.5% of coagulase-negative *Staphylococci* were resistant to methicillin, 20.5% of coagulase-negative staphylococci strains were characterized by MLSB (macrolides, lincosamides, and type B streptogramins) resistance mechanism, and 31.5% had MSB (macrolides and type B streptogramins) resistance mechanism. Nine of the isolated strains showed multidrug resistance. The authors emphasized the need to eradicate multidrug-resistant strains and determine the genome of the strain [96]. It is also important to note the underestimated role of CRS in the development of nosocomial infections in orthopedics, cardiac surgery, and transplantation. It is essential to consider and implement early identification and surveillance of antibiotic-resistant strains in all laboratories. Personalized treatment and appropriate epidemiological procedures will help control the spread of multidrug-resistant strains and prevent nosocomial infections.

Numerous hypotheses have been put forward to explain the occurrence of difficult-to-treat bacterial species in patients with CRS, including bacterial biofilm formation, intracellular survival of pathogens, and immune response to *S. aureus* superantigens. None of the hypotheses has been confirmed to date. Most resistance genes are localized on mobile genetic elements, allowing them to be easily transferred between organisms by horizontal gene transfer. Biofilm makes it difficult for the antibiotic to reach its site of action [9]. Biofilm formation in the sinus and nasal mucosa is associated with disease recurrence, poor response to treatment, and unfavorable surgical outcomes. Many species of bacteria and fungi can live in a single biofilm. Bacterial biofilms are detected in the sinus mucosa in as many as 80% of patients with CRS. Common species identified in biofilms include *S. aureus*, *S. pneumonia*, *P. aeruginosa*, *H. influenza*, *Acinetobacter* spp., *Proteus mirabilis*, and *Enterobacter* spp. coagulase-negative *Staphylococci*. Biofilms also occur on healthy sinus mucosa and their presence does not mean they cause mucositis. However, in the context of CRS, there are several possible mechanisms by which biofilms can have a pro-inflammatory effect, cause ciliary dysfunction, and inhibit mucociliary clearance [15].

Antibiotics can have a detrimental effect on commensal organisms and thus destabilize the native microflora. The lack of protective effect of commensal microorganisms can result in increased susceptibility to colonization by pathogenic bacteria. Destabilization of the bacterial community and reduced microbial diversity as a result of antibiotic treatment can predispose patients to secondary infections and the development of chronic inflammation: colitis caused by *Clostridium difficile* and post-antibiotic diarrhea [4,97]. Changes in the composition of microbial communities caused by exposure to antibiotics can take several months for the microbiome to rebuild [97]. In a study by Liu et al., the sinus microflora of six patients with CRS was compared before and after antibiotic treatment [98]. The specimens were collected from the maxillary sinuses in all patients. The material was then analyzed by 16S rRNA sequencing. Before treatment, a broad spectrum of sinus microflora was identified, and a uniform microbial profile was not demonstrated after the treatment. Responses to therapy varied widely, with changes in microflora composition varying from patient to patient. It was found that patients were more often colonized with strains that were less sensitive to the prescribed antibiotics. A significant reduction in bacterial diversity was also observed after antibiotic therapy. Nevertheless, it is unclear whether the effects were secondary to antibiotic use or after the disease had resolved [98].

A cross-sectional study by Feazel et al. found that antibiotic use, asthma, and previous surgeries affect the sinus microbiome [99]. Antibiotics and asthma correlated with a significant reduction in bacterial diversity and increased abundance of *S. aureus* strains, while prior surgery was associated with a reduced bacterial population. Such observations support the hypothesis that long-term, repeated administration of antibiotics can reduce the diversity of the microbial community and lead to the emergence of a few dominant bacterial species. Further research is needed to determine whether drugs, surgery and/or the natural course of time are responsible for such changes in microbial composition and diversity. Numerous reports have shown similar effects of ENT surgery on fungal populations [99].

## 7. Treatment with Probiotics

Given the current improved understanding of the potential role of the sinus microbiome in maintaining sinus health, antibiotic therapy may disrupt the dynamics of the bacterial community. Probiotics or prebiotics (non-viable food components that modulate the microflora for the benefit of the host) can be used as an alternative or supplement to antibiotic therapy. Probiotic supplementation, as a new therapeutic modality, aims to competitively inhibit pathogens or facilitate recolonization of the sinuses by desirable commensal bacteria. Probiotics can be administered orally to induce systemic immunity in the gut or topically to modulate the local immune response. Probiotics involve the administration of live microorganisms in sufficient quantities to directly induce beneficial physiological effects in the host. The mechanisms by which such microorganisms provide protection against pathogen invasion are multiple and typically involve modification of host immunity through the intestinal ecosystem. Commensal bacteria have been shown to enhance mucosal barrier integrity, induce the secretion of antimicrobial peptides, and competitively inhibit bacterial adhesion and colonization [29].

Understanding microbial interactions will be crucial to establishing the function of the microbial community in CRSs and implementing new therapeutic strategies. The interaction between *S. aureus* and *Corynebacterium* in the nasal cavity of a healthy human has been studied, and it has been shown that *Corynebacterium* sp. is involved in both mutual and inhibitory interactions with *S. aureus* [5]. A theory that the products (i.e., bacteriocin, lactic acid) produced by these microorganisms can impede excessive pathogen growth through competitive inhibition has also been proposed. *P. acnes* strains identified in 80% of control patients secrete bacteriocin, which not only has antibacterial and antifungal properties but also modulates the innate immune response to infection. This lends credence to the hypothesis that patients with a richer baseline sinus and nasal microbiome may be less susceptible to infection. 

Numerous internal and acquired immune responses are involved in host defense in the sinus cavities. The literature data confirm that systemic intake of probiotics increases the lymphocyte-interferon and interleukin-2 response and shifts the Th lymphocyte balance toward an increased Th1:Th2 ratio. Since allergic diseases, asthma, and CRSwNP have been linked to inadequate Th2 response, such effects may also help protect against CRSs. A reduction in T-regulatory cells and the presence of immunoglobulin E for the *S. aureus* superantigen have also been reported in CRSwNP [28]. Oral intake of probiotic microorganisms may preferentially enhance Th1 and T regulatory responses, which, in turn, may help offset the excess Th2 activity characteristic of these conditions [28].

The use of probiotics in the prevention and treatment of gastrointestinal diseases has long been known. Studies analyzing the effect of probiotic administration on the treatment of non-intestinal ailments have been undertaken. Probiotics have proven effective in treating atopic dermatitis and pollen allergies. Patients with allergic rhinosinusitis who received *Lactobacillus paracasei* reported improved quality of life compared to the control group [100]. *Streptococcus salivarius*, a non-pathogenic oral microbe that produces bacteriocin, reduces the colonization of bacteria involved in upper respiratory tract infections and is used as a probiotic. Oral preparations of *S. salivarius* are already available on the market. Such therapies can also be extrapolated to the sinuses, using commensal organisms as probiotics to inhibit colonization and overgrowth of pathogens. Similarly, Abreu et al. showed that topically applied *L. sakei* protects against *C. tuberculostearicum* in a mouse model of CRS [100]. Uehara et al. also showed that intranasal administration of *Corynebacterium* species was able to eliminate *S. aureus* in 71% of patients [101]. In a randomized, controlled trial involving 77 patients with CRS, there were no significant differences in the incidence of symptoms between patients who received the oral probiotic *Lactobacillus rhamnosus* R0111 (*n* = 39) for four weeks compared to placebo (*n* = 38). It should be noted that the study included and analyzed a total of both CRSsNP patients and CRSwNP patients, so it is unclear whether probiotics had different effects on different subtypes of CRS [28].

The broad spectrum of microbial profiles in CRSs, the lack of a uniform post-treatment microbiota, and highly individualized responses to treatment make it challenging to develop generalized therapeutic protocols for CRSs using probiotics. Different subtypes of CRS may also have divergent sinus microbiomes that contribute to a variable response to treatment, highlighting the potential futility of finding a universal antimicrobial treatment regimen. The initial challenge in probiotic treatment of CRS is choosing the right microorganism for a particular subtype of the disease, as the immunoregulatory effect is likely to be strain- and concentration-dependent. Extensive research is therefore needed to identify the determinants of virulence and to investigate metabolic, enzymatic, or hemolytic activity that may be potentially harmful to the host. In addition, the topical probiotic must also adhere to sinus and nasal tissue and must not have any harmful effects on other microflora residing on the skin. If probiotic use proves to be effective, the coming years may see a paradigm shift in the treatment of CRS away from attempts to eliminate bacteria toward restoring the natural ecology of the sinuses. Although probiotic therapies seem promising, much larger studies are needed to establish their true role in treating CRS [29].

## 8. Biological Treatment

Previously, treatment for CRS has included the chronic use of intranasal or oral anti-inflammatory agents, nasal irrigation with 0.9% sodium chloride solution, and surgical treatment. Despite the availability of increasingly effective and safer intranasal glucocorticosteroids and continuous improvement in surgical techniques, satisfactory results were not achieved in some patients [102].

The 2020 EPOS resolutions considered the pathophysiology of the inflammatory response in the taxonomical classification of CRS. Based on the mechanism of inflammation, two CRS endotypes were distinguished: type 2 and non-type 2 inflammation. Endotype 2 is associated with eosinophilic inflammation and a Th2-dependent inflammatory reaction involving IL-4, IL-5, and IL-13, and an increase in IgE levels. The non-type 2 endotype is neutrophilic inflammation. Patients with endotype 2 inflammation were much more likely than those with non-type 2 endotype inflammation to be resistant to therapies, and they tended to relapse [103].

In laryngology, attempts have been made for many years to assess the effectiveness of monoclonal antibodies in treating CRS. So far, monoclonal antibodies have been used primarily for treating cancer, psoriasis, atopic dermatitis, asthma, cystic fibrosis, viral hepatitis, and rheumatoid arthritis [103]. The EPOS 2020 working group determined that a CRS patient with bilateral polyps who has undergone surgery or has been disqualified from surgery and meets three of the following criteria is eligible for biological treatment: documented presence of a type 2 inflammatory reaction, tissue eosinophilia ≥ 10 in the visual field; or -blood eosinophilia ≥ 250; or total IgE ≥ 100, with a need for or contraindication to systemic glucocorticosteroids use, ≥2 treatments per year or long-term (>3 months) use of glucocorticosteroids in low doses, significant deterioration of quality of life, sino-nasal outcome test ≥ 40, significant loss of smell, anosmia, co-occurrence of asthma, asthma requiring regular treatment with inhaled glucocorticosteroids [103].

Biological therapies are different from traditional therapies. Biological drugs are monoclonal antibodies that help inhibit abnormal immune system reactions. Biological drugs currently used in CRS include monoclonal antibodies directed against the receptors for interleukin 4 (dupilumab), interleukin 5 (benralizumab), and those directly blocking interleukin 5 itself (mepolizumab, reslizumab) or free immunoglobulin E (omalizumab). In 2019, the Food and Drug Administration and the European Medicines Agency approved dupilumab for the treatment of CRSwNP. The costs of biological agent treatments are high. In Poland, no biological drugs are currently included in the list of reimbursed preparations for CRS therapy [103].

Dupilumab is one of the newest promising biological drugs; it is a monoclonal antibody directed against the α subunit of the interleukin-4 receptor (IL-4Rα, common to IL-4 and IL-13 receptors) [104]. Therefore, blocking IL-4R alpha with dupilumab inhibits both IL-4 and IL-13 signaling simultaneously [103]. The drug has been used for several years with good clinical results in treating atopic dermatitis and asthma with T2 inflammation, in Poland, as well as in treating CRSwNP in many other countries [104]. Meta-analyses have shown that dupilumab significantly reduces the level of chemokines and cytokines, improving the quality of life in patients with chronic sinusitis with nasal polyps complicated by asthma [105]. At the same time, possible side effects were demonstrated: reactivation of the herpes virus, conjunctivitis, and reactions at the injection site [106]. After discontinuing the drug, the effect of treatment gradually decreases, and antibodies against the drug may also appear [103]. Other biological drugs registered in Poland for the treatment of CRSwNP include mepolizumab and omalizumab [102].

Omalizumab inhibits the IgE-induced inflammatory cascade. Reducing the concentration of IgE is possible by blocking the receptors of these cytokines on B lymphocytes with monoclonal antibodies or binding free IgE with genetically engineered immunoglobulins directed against them. Omalizumab is a drug approved for use in Europe and the USA in combination therapy with intranasal steroids in adult patients with severe chronic sinusitis with nasal polyps in whom intranasal steroid therapy does not produce therapeutic effects. In Poland, the drug is reimbursed under the drug program, among others, for asthma. It is not refunded in the case of CRS. Contraindications to the use of the drug include low IgE levels, high patient weight, age below six years, and parasitic diseases. Side effects of omalizumab include headaches, joint pain, abdominal pain, dizziness, fever, and injection site reactions. Rare but serious complications result from the formation of immune complexes: cardiovascular events, stroke, severe idiopathic thrombocytopenia and serum sickness [107], hypereosinophilia and eosinophilic granulomatosis with polyangiitis, and lymphoblastic lymphoma [108,109]. Clinical trials have shown improvement in nasal problems, such as nasal blockage, rhinorrhea, and impaired sense of smell, as well as a reduction in the size of nasal polyps and the number of inflammatory lesions in the sinuses [104]. The results of other meta-analyses range from improvement in nasal symptoms [110] to no reduction in serum IgE levels [111].

IL-5 is produced primarily by antigen-stimulated Th2 lymphocytes but also by mast cells. IL-5 influences the differentiation and maturation of precursor cells in the bone marrow towards eosinophils. The highest concentration of IL-5 was found in patients with nasal polyps, non-allergic asthma and aspirin hypersensitivity accompanied by eosinophilia [103].

Mepolizumab is an anti-IL-5 monoclonal antibody. It inhibits eosinophilic inflammation by interfering with the binding of IL-5 to receptors expressed on eosinophils and basophils [112]. In 2021, the FDA approved mepolizumab for the treatment of chronic sinusitis with nasal polyps. In a 2011 study, compared to a placebo-treated control group, the size of nasal polyps in patients in the mepolizumab-treated group was significantly reduced [113]. Another clinical study reported that 70% of patients with chronic sinusitis with nasal polyps still required surgical treatment despite receiving mepolizumab [114]. Common side effects of mepolizumab include headache and injection site reactions. No cases of allergic reactions have been reported. The availability of the drug is low for patients due to its high price [115].

Reslizumab is also a humanized anti-IL-5 antibody [103]. To date, one randomized clinical trial has been carried out to evaluate the effects of reslizumab in a small group of patients with chronic sinusitis with nasal polyps [116]. A reduction in nasal polyps compared to baseline values was confirmed, as well as a significant reduction in the number of eosinophils in the blood just 12 h after administration, and this effect lasted for eight weeks. Reslizumab has a good safety profile. The most frequently observed side effect was an upper respiratory tract infection. Increased blood creatine kinase levels and an anaphylactic reaction also occurred [103].

Benralizumab is a monoclonal antibody that binds to the IL-5 receptor. Clinical trial results showed that benralizumab significantly improved nasal symptoms and other clinical symptoms associated with chronic sinusitis with nasal polyps to varying degrees [117]. Benralizumab is expected to gain FDA approval in the next few years for the treatment of chronic sinusitis with nasal polyps [103]. Benralizumab has a good safety profile. The most common side effects are a cold and headache. Serious side effects are rare, such as cytokine release syndrome, mydriasis, and pneumonia [118]. Anaphylactic shock has also been reported to occur several hours after administration of the drug [104].

Other biologics targeting IL-25, IL-33, and thymic stromal lymphopoietin (TSLP) are currently in clinical trials [104]. Studies so far have not confirmed that biological agents can replace surgical treatment. Therefore, biologics should be used as an additional treatment for chronic sinusitis with nasal polyps or as an option for patients who cannot undergo surgical treatment [104].

Biological treatment requires systematic monitoring of the patient’s condition. The first assessment is made after six months of therapy. If no improvement is observed based on tests, treatment should be discontinued, and surgical or other biological treatment should be considered [102].

High drug prices make it difficult for patients to access treatment options with biological drugs. The use of biomarkers to classify and identify the targets of biological drugs will lead to the identification of more precise treatment options. More research based on new therapeutic targets and carefully selected biological agents is necessary to confirm their safety and therapeutic effectiveness. For biological agents to become a new treatment for most patients, in addition to traditional drugs and surgery, cost reduction will be necessary [104,119].

## 9. Conclusions

CRS is a major public health problem and has a huge impact on quality of life. CRS is characterized by persistent inflammation, a dysregulated immune response and interactions with microorganisms, which together cause epithelial barrier dysfunction, tissue remodeling, and clinical symptoms. Mucociliary function is a key host defense mechanism that removes inhaled particulate matter. Bacterial colonization, along with impaired mucociliary clearance, plays a significant role in initiating or sustaining the inflammatory process in CRS. Age-related physiological and pathological changes on the surface of the nasal mucosa may alter the host immune response and may be strongly associated with changes in the bacterial microbiota of patients with CRS.

Human studies have shown that the CRS microbiome is characterized by a loss of diversity compared to healthy controls, indicating an opportunity for pathogen development. Disruption of the commensal bacteria interaction with the local immune system appears to be a critical determinant in CRS progression. A number of inflammatory pathways and host defense elements are altered in CRS, which may contribute to the observed differences in the microbiome. Nevertheless, one still needs to determine whether the changes observed in the nasal microbiome are a cause or effect of this chronic inflammatory disease.

To date, research on CRS has been limited due to poor access to the tissue being studied, the complexity of sinus and nasal physiology, the lack of available biomarkers, and the lack of useful animal models. Further analyses are needed to characterize the virulence profiles of known and newly identified microorganisms, their role in the pathophysiology of CRS, and their relationship to disease severity. In addition, extensive research is required to explain the ecological and environmental pressures that affect the sinus microbiome and how specific microbial species and/or strains may affect health or disease. Better defining the complex dynamics between the sinus-dwelling microflora and the host immune system is also crucial for guiding future medical therapy. CRS treatment will become more tailored to the pathophenotype as our knowledge of the complex dynamics of the microbial community expands. Specific probiotic and prebiotic therapies should address microbiota disorders characteristic of individual patients.

Future research should focus on the development of genomics, transcriptomics, and metabolomics studies, which will enable a better understanding of how microorganisms function in health and disease and open up many new opportunities in CRS treatment and diagnostics. In addition, we believe that new diagnostic methods and personalized medicine, which is based on clinical, genetic, and environmental data unique to each patient, will facilitate the prevention and treatment of CRS.

## Figures and Tables

**Table 1:** Advantages and disadvantages of classical and molecular methods for detecting microbiota of CRS.

Techniques/Methods	Advantages	Disadvantages
Culture techniques	- Lower price; - The ability to conduct tests in smaller microbiology laboratories; - No need to purchase specialized; - Expensive equipment;	- Difficulties in identifying microorganisms present in small numbers in a test sample; - Difficulties in identifying microorganisms that require special culture conditions; - Difficulties in identifying microorganisms sensitive to environmental conditions (transportation); - Long waiting time for the result;
Molecular methods	- Fast (short waiting time for the result); - Detection of microorganisms present in small numbers in a sample; - The ability to study the interactions between microorganisms; - The ability to create databases for the studied microorganisms; - Higher sensitivity and specificity compared to classical techniques; - The ability to study changes in the genome and the presence (expression) of microbial antibiotic resistance genes.	- 16S rRNA gene sequencing measures the total or relative abundance of bacterial DNA—PCR does not distinguish between actively growing, dormant, or dead biomass; - Contamination problems; - The need to transport and store the material for testing under special conditions (extremely low temperature); - Higher price; - Tests are performed in selected specialized laboratories with appropriate equipment.

## Data Availability

Not applicable.

## Supplementary Materials

The following supporting information can be downloaded at: https://www.mdpi.com/article/10.3390/ijms25063201/s1.
