# Supplementary material for: Chronic Rhinosinusitis—Microbiological Etiology, Potential Genetic Markers, and Diagnosis
Source: Int J Mol Sci. 2024 Mar 11;25(6):3201. doi: 10.3390/ijms25063201 (PMC10970433; doi:10.3390/ijms25063201)
Supplement: Supplementary file 1 [file ijms-25-03201-s001.zip › ijms-2874605-supplementary.pdf]

## Supplementary materials

# Chronic Rhinosinusitis—Microbiological Etiology, Potential Genetic Markers, and Diagnosis

**Michał Michalik [1,†] and Beata Krawczyk [2,*,†]**

[1] Medical Center MML, Bagno 2, 00-112 Warsaw, Poland; m.michalik@mml.com.pl
[2] Department of Biotechnology and Microbiology, Faculty of Chemistry, Gdańsk University of Technology, G. Narutowicza 11/12, 80-233 Gdańsk, Poland
* Correspondence: beata.krawczyk@pg.edu.pl
† These authors contributed equally to this work.

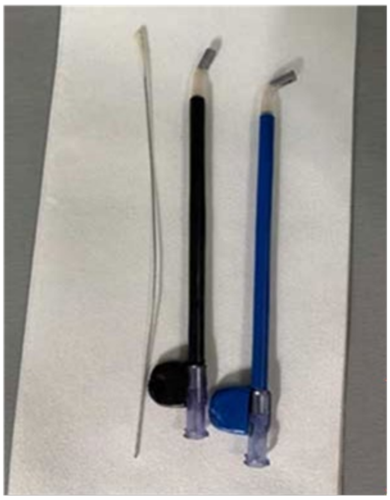

Figure S1. Three cannulas with different angles, contoured at 15, 45 and 120 degrees, respectively for collecting microbiological samples of high-diagnostic specimens from diseased paranasal sinuses.

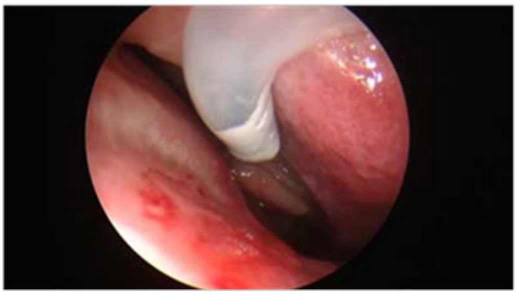

Figure S2. Use of an angled cannula during the procedure of collecting microbiological samples.
